# β-Dystroglycan Restoration and Pathology Progression in the Dystrophic *mdx* Mouse: Outcome and Implication of a Clinically Oriented Study with a Novel Oral Dasatinib Formulation

**DOI:** 10.3390/biom11111742

**Published:** 2021-11-22

**Authors:** Paola Mantuano, Brigida Boccanegra, Elena Conte, Michela De Bellis, Santa Cirmi, Francesca Sanarica, Ornella Cappellari, Ilaria Arduino, Annalisa Cutrignelli, Angela Assunta Lopedota, Antonietta Mele, Nunzio Denora, Annamaria De Luca

**Affiliations:** 1 Section of Pharmacology, Department of Pharmacy—Drug Sciences, Orabona 4—Campus, University of Bari “Aldo Moro”, 70125 Bari, Italy; paola.mantuano@uniba.it (P.M.); brigida.boccanegra@uniba.it (B.B.); elena.conte@uniba.it (E.C.); michela.debellis@uniba.it (M.D.B.); santa.cirmi@uniba.it (S.C.); francesca.sanarica@uniba.it (F.S.); ornella.cappellari@uniba.it (O.C.); antonietta.mele@uniba.it (A.M.); 2 Section of Pharmaceutical Technologies, Department of Pharmacy—Drug Sciences, Orabona 4—Campus, University of Bari “Aldo Moro”, 70125 Bari, Italy; ilaria.arduino@uniba.it (I.A.); annalisa.cutrignelli@uniba.it (A.C.); angelaassunta.lopedota@uniba.it (A.A.L.); nunzio.denora@uniba.it (N.D.)

**Keywords:** Duchenne muscular dystrophy, preclinical study, *mdx* mouse, dasatinib, cyclodextrin, oral formulation, pediatric age

## Abstract

ROS-activated cSrc tyrosine kinase (TK) promotes the degradation of β-dystroglycan (β-DG), a dystrophin-glycoprotein complex component, which may reinforce damaging signals in Duchenne muscular dystrophy (DMD). Therefore, cSrc-TK represents a promising therapeutic target. In *mdx* mice, a 4-week subcutaneous treatment with dasatinib (DAS), a pan-Src-TKs inhibitor approved as anti-leukemic agent, increased muscle β-DG, with minimal amelioration of morphofunctional indices. To address possible dose/pharmacokinetic (PK) issues, a new oral DAS/hydroxypropyl(HP)-β-cyclodextrin(CD) complex was developed and chronically administered to *mdx* mice. The aim was to better assess the role of β-DG in pathology progression, meanwhile confirming DAS mechanism of action over the long-term, along with its efficacy and tolerability. The 4-week old *mdx* mice underwent a 12-week treatment with DAS/HP-β-CD10% dissolved in drinking water, at 10 or 20 mg/kg/day. The outcome was evaluated via in vivo/ex vivo disease-relevant readouts. Oral DAS/HP-β-CD efficiently distributed in *mdx* mice plasma and tissues in a dose-related fashion. The new DAS formulation confirmed its main upstream mechanism of action, by reducing β-DG phosphorylation and restoring its levels dose-dependently in both diaphragm and gastrocnemius muscle. However, it modestly improved in vivo neuromuscular function, ex vivo muscle force, and histopathology, although the partial recovery of muscle elasticity and the decrease of CK and LDH plasma levels suggest an increased sarcolemmal stability of dystrophic muscles. Our clinically oriented study supports the interest in this new, pediatric-suitable DAS formulation for proper exposure and safety and for enhancing β-DG expression. This latter mechanism is, however, not sufficient by itself to impact on pathology progression. In-depth analyses will be dedicated to elucidating the mechanism limiting DAS effectiveness in dystrophic settings, meanwhile assessing its potential synergy with dystrophin-based molecular therapies.

## 1. Introduction

Duchenne muscular dystrophy (DMD) is a rare neuromuscular X-linked disease belonging to a group of disorders known as dystrophinopathies. The pooled global and birth prevalence of DMD is 7.1 (95% CI: 5.0–10.1) and 19.8 (95% CI: 16.6–23.6) per 100,000 males, respectively [1,2]. DMD is caused by mutations in the dystrophin gene (DMD, *300377) that lead to the absence of dystrophin, a cytoskeletal protein, or to its structural defects which impair stability and function [3]. The lack of functional dystrophin causes the disassembly of the dystrophin-glycoprotein complex (DGC), with the consequent reduction of one its crucial transmembrane components, β-dystroglycan (β-DG). In normal myofibers, β-DG provides a link between dystrophin and extracellular laminin, ensuring sarcolemmal stability. In addition, β-DG is also involved in signaling, via the interaction with specific intracellular partners, under the control of post-translational modifications, such as the phosphorylation of tyrosine residues [4].

In dystrophin-deficient myofibers, the primary structural defect brings to a progressive muscle degeneration and aberrant signaling during contraction [3], as well as weakness, muscle loss, oxidative stress, and inflammation [2,5]. To date, no resolutive cure is available for DMD and the most effective treatment regimen aimed to relieve symptoms and slow disease progression entails the use of anti-inflammatory glucocorticoids [6].

In physiological conditions, mainly due to contractile activity and high oxygen consumption, skeletal muscles produce moderate and constant levels of reactive oxygen species (ROS). These are balanced by detoxification systems to preserve redox homeostasis [7]. In dystrophic myofibers, the lack of dystrophin protein can directly induce ROS production, which contributes to sarcolemmal damage, mitochondrial Ca^2+^ overload, and pro-inflammatory NF-κB pathway activation [8]. In addition, also muscle function can be directly affected by oxidative stress. For instance, the impairment of fast-twitch muscles contractile function in dystrophic *mdx* mice, has been recently correlated with a defect in selenoprotein S (*Seps1*), an endoplasmic reticulum oxidoreductase involved in ROS neutralization [9].

Among redox-sensitive proteins, Src tyrosine kinase (TK) is of sure interest as potential drug target in DMD. In healthy muscles, Src-TK is involved in cell division and growth, as well as in the stabilization of neuromuscular junctions [10]. Src-TK is overexpressed in dystrophic myofibers and can be overactive due to excessive ROS production [11,12]. In the absence of dystrophin, ROS-activated cSrc-TK contributes to both β-DG phosphorylation and degradation; this event reinforces damaging signaling in DMD, in relation to the direct role of β-DG in mechano-transduction [13]. In addition, cSrc TK can affect autophagy, with a further worsening of the pathogenic cascade [14]. Therefore, the inhibition of Src-TK could be a feasible strategy for DMD therapeutic treatments. In fact, Src-TK may act on both upstream—via β-DG degradation—and downstream events, leading to muscle wasting. Dasatinib (DAS) is a double inhibitor of kinase proteins, including proto-oncogene Src-TK family kinases. Nowadays, DAS is used as first-choice oral drug in the treatment of chronic myeloid leukemia (CML) for patients who are resistant or intolerant to imatinib. Lipscomb and colleagues showed that DAS decreases the degree of β-DG tyrosine phosphorylation and increases the relative levels of non-phosphorylated β-DG in the dystrophic *sapje zebrafish.* Furthermore, DAS treatment resulted in an improved physical appearance of *sapje zebrafish* musculature and an increased swimming ability of treated fish compared with controls [15]. In addition, in November 2017 DAS was approved by the Food and Drug Administration (FDA) for the treatment of pediatric patients with CML in the chronic phase, paving the way for a possible repurposing in DMD boys. Recently, our research group performed a proof-of-concept 4-week treatment with subcutaneous administration of DAS in treadmill exercised *mdx mice*. Interestingly, a significant increase in muscle levels of both total and non-phosphorylated β-DG was found, although functional and histological markers of pathology were minimally, if at all, ameliorated [16]. This limited efficacy on functional parameters, in spite of a confirmed mechanism of action, opened the main question about insufficient dosing and/or duration treatment to exert appreciable impact on disease progression. In the view to address possible pharmacokinetic issues and feasibility of a long-term dose-dependent study, a novel formulation of DAS was obtained in complex with hydroxypropyl-beta-cyclodestrin (HP-β-CD). A short-term pilot study in wild-type (WT) mice showed that DAS/HP-β-CD has a good tolerability and oral bioavailability, with proper plasma and skeletal muscle drug exposure [17]. This new DAS/HP-β-CD formulation pushed us to perform a dose-related three months treatment in *mdx* mice, to better assess the role of β-DG in pathology progression and the clinical promise of DAS in DMD. To this aim, the study was constructed according to the main guidelines required for clinically oriented preclinical tests [18,19,20] in order (a) to confirm the mechanism of action over the long term and in relation to DAS administration in a pediatric-suitable oral formulation; (b) to assess efficacy on clinically relevant readouts; (c) to envisage any potential limitations either in terms of efficacy or safety.

## 2. Materials and Methods

All the experiments were conducted in conformity with the Italian Guidelines for Care and Use of Laboratory Animals (D.L.116/92) and with the European Directive (2010/63/UE). The study was approved by the National Ethics Committee for Research Animal Welfare of the Italian Ministry of Health (authorization no. 815/2017-PR and 1119/2020-PR). All the established experimental protocols conform to the standard operating procedures (SOPs) for preclinical tests in *mdx* mice, available on the TREAT—NMD website http://www.treat-nmd.eu/research/preclinical/dmd-sops/ (accessed on 27 September 2021) [19,20]. Animal studies have been reported in compliance with the ARRIVE guidelines [21].

### 2.1. In Vivo Procedures

#### 2.1.1. Animal Groups, Drug Formulation, and Treatment Schedule

A total of 24, 4–5 week-old, male *mdx* mice (C57BL/10ScSn*Dmd^mdx^*/J) and 6 age- and sex-matched control wild type mice (C57BL/10ScSnJ) were purchased from The Jackson Laboratory (USA, distributed by Charles River, Calco, Italy). All mice were acclimatized in the animal facility before starting the experimental protocol. Animals were housed in suitable cages (3–5 mice per cage) based on mean body mass (BM), in a single room where appropriate conditions of temperature (22–24 °C), humidity (50–60%), and light/dark cycle (12 h/12 h) were constantly maintained for the entire duration of the study. After acclimatization, *mdx* mice were randomly assigned to each experimental cohort (n = 7–9 mice per group). Mice were maintained on a controlled diet, with a 4–5 g/mouse daily amount of complete chow (VRF1 standard pelleted diet, Charles River Laboratories) [22] and constantly, non-invasively monitored for health and well-being; none of the experimental groups showed signs of pain or distress or macroscopic alterations of vital functions.

Dasatinib (DAS; BMS-354825, Selleckchem, Houston, TX, USA distributed by Aurogene, Rome, Italy) was complexed with hydroxypropyl-beta-cyclodextrin (HP-β-CD, Farmalabor Srl, Canosa di Puglia, BT, Italy) to obtain two different doses (10 and 20 mg/kg) for administration in drinking water, as previously described [17]. The drug was prepared once a week, and the administered doses were adjusted according to mice body mass and water consumption. Daily water intake was obtained dividing the weekly amount of water consumed per cage, by the number of weekdays and by the number of mice allocated in that cage; then, it was normalized to their mean BM.

According to the results obtained by our proof-of-concept study [16], the duration of the treatment was chosen to be 12 weeks. To avoid introducing any bias, all experimental procedures, as well as data collection and analysis, were carried out by blinded experimenters.

#### 2.1.2. Forelimb Grip Strength Test and Treadmill Exhaustion Test

Forelimb force was assessed on a weekly basis by means of a grip strength meter (Columbus Instruments, Columbus, OH, USA) according to SOPs for *mdx* mice (TREAT—NMD SOP (ID) Number: DMD_M.2.1.001). Maximal force, both absolute (expressed in kg force, KGF) and normalized to BM (in KGF/kg), obtained from five repeated measurements per mouse, was used for data analysis [5,22,23,24].

At the beginning (T0) and at the end (T12) of the experimental protocol, all mice underwent an acute exercise resistance test on a horizontal treadmill (Columbus Instruments) to assess in vivo fatigability (TREAT—NMD SOP (ID) Number: DMD_M.2.1.003). During the test, each mouse was made to run until exhaustion (i.e., the inability to re-start the running after a 20 s pause) and the total distance run (in meters) up to that time was calculated and used for data analysis [16,22,23,25,26].

#### 2.1.3. Isometric Plantar Flexor Torque

At T12, the isometric torque produced by hind limb plantar flexor muscles (i.e., gastrocnemius, soleus, plantaris) was assessed in vivo in anesthetized mice from each cohort by means of the 1300A *3-in-1 Whole Animal Test System* (Aurora Scientific Inc.—ASI, Aurora, ON, Canada). Inhalation anesthesia (≈3% isoflurane in an induction chamber, then ≈2% isoflurane via nose cone for maintenance, both with 1.5 L/min O_2_) was delivered by using an anesthetic vaporizer (Harvard Apparatus Fluovac and Datex Ohmeda Isotec 4, Holliston, MA, USA) with an oxygen concentrator (LFY-1-5A, Longfei Group Co., Wenzhou, China; distributed by 2Biological Instruments, Besozzo, VA, Italy). After prepping the skin of the right hind limb by removing hair and cleaning, the animal was placed supine on a temperature-controlled platform (mod. 809B, ASI) at 36 °C, with the right foot taped to a footplate connected to a dual-mode servomotor (mod. 300C-LR, ASI), forming a 90° angle with the hind limb secured at the knee. Contractions were elicited via percutaneous electrical stimulation of the sciatic nerve, through a pair of needle electrodes (Chalgren Enterprises Inc., Gilroy, CA, USA) connected to a high-power, bi-phase stimulator (mod. 701C, ASI), in turn controlled by a data acquisition signal interface (mod. 604A, ASI) and by ASI Dynamic Muscle Control software (DMCv5.415). Initial twitches, evoked with 0.2 ms single square wave pulses, were used to adjust the current (from 25 to 40 mA) to maximize torque production. Then, a series of isometric contractions was recorded at increasing frequencies (200 ms pulses at 1, 10, 30, 50, 80, 100, 120, 150, 180, 200 Hz, one every 30 s). Plantar flexor torque obtained at each frequency (N*cm) was calculated via ASI Dynamic Muscle Analysis software (DMAv5.201). Then, torque values were normalized to each mouse BM (N*mm^3^/kg) and used to construct torque—frequency curves [16,24,27].

#### 2.1.4. Diaphragm Ultrasonography

Ultrasound imaging of the diaphragm (DIA) muscle was also performed at T12 on all mice groups, by using the ultra-high frequency ultrasound biomicroscopy system Vevo^®^ 2100 (VisualSonics, Toronto, ON, Canada). Prior to each imaging session, each animal, put under isoflurane anesthesia, was placed on a thermostatically controlled platform (37 °C) in dorsal decubitus position and adequately prepared for the experiment to allow optimal image acquisition. Both mouse and probe were properly positioned as described in full detail in previous work [26]. Image acquisition was performed in mono-dimensional (M-Mode) and bi-dimensional (B-Mode), by using a probe operating at a frequency of 21 MHz with a lateral and axial resolution of 165 and 75 μm, respectively. DIA movement amplitude was measured in M-Mode during normal breathing cycles on the left side, which provides less variability in measurements for all experimental groups. The amplitude during each inspiration (positive deflection) was measured as the distance (in mm) between the baseline and the peak of contraction. For each mouse, DIA amplitude was calculated as the mean value obtained from 3–5 measurements [26,28]. Images acquired in B-Mode were used to evaluate DIA echodensity, which was measured using ImageJ^®^ software by creating a grey scale analysis histogram on the entire outlined DIA section of a constant dimensions of 4514.0 ± 17.6 pixels. In particular, for each mouse, DIA echodensity was obtained as the main value obtained from 4 frames of the same acquisition drawing the region of interest (ROI) in the same area of the DIA. Variations in echodensity were expressed as percentage differences between wt and *mdx* groups of the mean echo intensity of the pixels included in the outlined area. Data sets were obtained by repeated, independent analyses to avoid intra- and inter-variability, and multiple analyses consistency and reproducibility was ensured as described in previous work [26].

### 2.2. Ex Vivo Procedures

#### 2.2.1. Sample Collection, Processing, and Storage

At the end of the 12th week of treatment, the ex vivo experimental phase started. Due to the time-consuming nature of functional ex vivo experiments on isolated muscles, a maximum of 2–3 animals per day were sacrificed. This required an additional experimental time window of 2–3 weeks. Mice continued to be treated until the day of sacrifice. For this reason, T12 was considered as the final time point for in vivo data statistical analysis. Plus, to avoid any bias due to different treatment exposure, mice from different cohorts were equally distributed over time during ex vivo experiments [5,16].

Each mouse was anesthetized via intraperitoneal (i.p.) injection with a cocktail of ketamine (100 mg/kg) and xylazine (16 mg/kg). If required, an additional lower dose of ketamine alone (30 mg/kg) was injected to ensure longer and deeper sedation. Several tissues were harvested from each mouse in this phase, following international guidelines, also to fulfil the 3Rs requirements about ethical animal use [19].

Both right and left hind limb tibialis anterior (TA), extensor digitorum longus (EDL), quadriceps (QUAD), gastrocnemius (GC), and soleus (SOL) muscles, as well as vital organs (liver, heart, kidneys, spleen, and brain) were isolated and weighed for a gross examination of drug toxicity and/or effects. Right after heart removal, also DIA muscle was collected from each animal.

A section of right hemi-diaphragm (from the ribs to the central tendon domain), and EDL muscle of the left limb were carefully prepared for isometric and eccentric contraction recordings, as described later on (Section 2.2.4). Another section of right hemi-diaphragm (only costal muscle domain) and right GC muscle were snap frozen in N_2_ and stored at −80 °C until further processing for Western blot experiments. In parallel, left hemi-diaphragm (costal muscle domain) and GC muscle of the left hind limb were embedded in a small amount of Tissue-Tek O.C.T. (Bio-Optica, Milan, Italy), immersed in isopentane cooled with liquid nitrogen (N_2_) for 60 s, and then stored at −80 °C until being further processed for histology. Both QUAD muscle of the left hind limb and liver were snap frozen in N_2_ and stored at −80 °C for further PK analysis. Blood was obtained by cardiac puncture with a heparinized insulin syringe and collected in heparinized tubes (Heparin Vister 5000 U.I./mL). Within 30 min after collection, platelet-poor plasma was obtained after two consequential centrifugation steps (20 min, 2000× *g*, 4 °C; 10 min, 10,000× *g*, 4 °C), and used fresh to measure creatine kinase (CK) and lactate dehydrogenase (LDH) by spectrophotometry; an aliquot was stored at −80 °C until PK analysis was performed. In addition, the remaining harvested hind limb muscles and organs were snap frozen in N_2_ and stored at −80 °C in our mouse tissue repository for possible future analyses.

#### 2.2.2. Pharmacokinetic Analysis

The quantification of DAS/HP-β-CD 10% complex concentration was carried out in plasma, liver, and QUAD samples from mice group treated with each dose (10 and 20 mg/kg/day), in comparison to untreated *mdx* mice, used as negative controls. For sample preparation, an initial stock solution was prepared in MeOH (1 mg/mL); then, an intermediate stock solution at 2 µg/mL analyte was prepared in acetonitrile (ACN), to prepare the final working solution. A total of 5 µL of working solution were spiked in 45 µL of each plasma sample, to obtain the desired final concentrations. Then, 50 µL of samples and calibrants were precipitated in 150 µL of cold ACN containing 0.01 µg/mL of verapamil as internal standard. Each sample was vortexed, centrifuged, and then the supernatant was transferred into a 96-well plate and injected into the UPLC-MS/MS. QUAD and liver samples were homogenized in 1.5 and 3.5 mL of 20 mM ammonium formiate buffer, respectively, and processed as above described for plasma samples. The calibration range was from 0.5 to 2000 ng/mL for all the three matrices. All samples were analyzed via an ACQUITY UPLC system (Waters Corporation, Milford, MA, USA) coupled with an API 3200 Triple Quadrupole AB Sciex mass spectrometer (AB Sciex, Framingham, MA, USA).

#### 2.2.3. Protein Expression Analysis by Immunoprecipitation and Western Blot

Protein extractions and immunoblots to quantify the expression of β-dystroglycan (β-DG) and vinculin (VINC), detected as reference standard, were performed in DIA and GC muscles from *mdx* mice, either treated or untreated, and WT mice as internal control. Muscles were homogenized in an ice-cold buffer containing 20 mM Tris-HCl (pH = 7.4 at 4 °C), 1% (*v*/*v*), Triton X-100, 1% (*v*/*v*) NP-40, 2 mM MgCl_2_, 5 mM EDTA, 150 mM NaCl, 0.2 mM phenylmethylsulfonyl fluoride, 1 mM sodium ortovanadate (NaVO_4_), 10 mM NaF, and protease inhibitors (10 mg/mL leupeptin and 10 mg/mL pepstatin). Homogenates were centrifuged at 1000× *g* for 10 min at 4 °C and the supernatant was quantified via Bradford assay (Bio-Rad Protein Assay Kit I5000001, Bio-Rad, Hercules, CA, USA). For the determination of phospho-β-dystroglycan (p-β-DG), immunoprecipitation was performed prior to immunoblot analysis: 10 μg of mouse primary antibody anti β-DG (Ab), diluted in 200 μL of PBS-Tween-20, were added to dynabeads protein G (Life Technologies, Carlsbad, CA, USA) and incubated for 3 h at 4 °C on a roller for mixing. After three washes with PBS, 60 μg of protein extract (Ag) were added, and samples were incubated overnight at 4 °C on a roller for mixing. After three washes with PBS, the dynabeads-Ab-Ag complex was resuspended in 20 μL of elution buffer, added with 20 μL premixed NuPage LDS sample buffer and NuPage sample reducing agent (Life Technologies), and heated for 10 min at 70 °C. Protein content was again quantified by Bradford assay (Bio-Rad). For immunoblot analysis, 60 μg of protein were separated on a 12% SDS-PAGE and transferred onto a PVDF membrane for 7 min at 1.3 A—25 V (Trans-Blot^®^ Turbo™ Transfer System, Bio-Rad), and protein detection was conducted as described in previous work [16]. The following dilutions of primary antibodies were used: 1:200 mouse anti β-DG (Novocastra, Leica Biosystems Italia, Buccinasco, MI, Italy); 1:500 mouse anti p-β-DG (Abcam, Cambridge, UK) and 1:400 mouse anti vinculin (Sigma—Aldrich, St. Louis, MO, USA). An anti-mouse horseradish peroxidase-conjugated secondary antibody (1:5000 anti-mouse IgG, Sigma—Aldrich) was used. β-DG was detected after stripping the membrane with β-mercaptoethanol-containing stripping buffer (20% SDS, Tris-HCl 1 M pH = 6.8, β-mercaptoethanol 0.1 M) for 30 min at 50 °C. The membrane was washed in washing buffer (PBS 10%, Tween-20 0.1%), before being incubated with the next primary antibody. Densitometric analysis was performed using Image Laboratory Software (Bio-Rad), that allows the chemiluminescence detection of each experimental protein band to obtain the absolute signal intensity. The density volume was automatically adjusted by subtracting the local background.

#### 2.2.4. Isometric and Eccentric Contraction Recordings of Isolated Muscles

A strip of right hemi-diaphragm (no more than 4 mm wide) was cut from the excised muscle and then firmly tied at the rib and at the central tendon, while the EDL muscle from the left hind limb was securely tied with silk suture 6-0 (Fine Science Tools Inc., Foster City, CA, USA) at the proximal and distal tendons during dissection. Two loops were made with sutures at both ends, to place each muscle into a recording chamber containing 25 mL of isotonic Ringer’s solution (composition in mM: NaCl 148, KCl 4.5, CaCl_2_ 2.0/2.5, MgCl_2_ 1.0, NaH_2_PO_4_ 0.44, NaHCO_3_ 12.0, glucose 5.55; pH 7.2–7.4), continuously gassed with a mixture of 95% O_2_ and 5% CO_2_, and thermostatically maintained at 27 ± 1 °C (TREAT—NMD SOP (ID) Number: DMD_M.1.2.002). Specifically, the DIA strip was placed into a vertical muscle bath, with the central tendon fixed to a hook at the bottom of the chamber, and the rib fixed to a dual-mode muscle lever (mod. 300C-LR, ASI); similarly, EDL muscle was placed into a horizontal muscle bath (mod. 809B-25, ASI), with the proximal tendon fixed to a 300C-LR force transducer and the distal tendon fixed to a hook at the opposite side of the chamber. In each bath, electrical field stimulation was obtained by two axial platinum electrodes closely flanking the muscle, connected to a high-power bi-phase stimulator (for DIA: LE 12406, 2Biological Instruments; for EDL: mod. 701C, ASI). Each apparatus was equipped with a data acquisition signal interface (for both systems: mod. 604A, ASI) and software (for DIA: DMCv4.1.6; for EDL: DMCv5.415, ASI).

After equilibration (~30 min), muscle preparations were stretched to their optimal length (L_0_, measured with an external caliper), which is the length producing the maximal single contraction (twitch, Ptw) in response to a 0.2 ms square wave 40–60 mV pulse. Single twitch tension was calculated as the mean value from 5 twitches elicited by pulses of 0.2 ms, every 30 s. Tetanic contractions were elicited by applying trains of 2.0 ms pulses for 450 ms (DIA) or 350 ms (EDL), at increasing frequencies (10, 20, 40, 60, 80, 100, 120, 140, 180, 200 Hz), every 2 min. Maximal tetanic force (P0) was usually recorded at 140–180 Hz. Then, both muscles underwent a series of 10 eccentric contractions, every 30 s. Briefly, an initial 300 ms isometric contraction was elicited, followed by a stretch of 10% L_0_ at a speed of 1 L_0_ s^−1^ imposed for the last 200 ms. The progressive decay in isometric force at 5th and 10th pulses was calculated as the percentage of reduction in force vs. the 1st pulse. Two tetanic stimuli (120 Hz, 500 ms) were elicited 4 and 30 min after the eccentric contraction protocol, to calculate the recovery from the stretch-induced force drop vs. the tetanic force registered before the protocol started, as well as muscles compliance to stretch.

Data were analyzed via ASI software DMAv3.2 for DIA and DMAv5.201 for EDL, to obtain Ptw and P0 values, then normalized to muscle cross sectional area according to the equation sP = P/(Mass/L_f_*D) where P is absolute tension, Mass is the muscle mass, D is the density of skeletal muscle (1.06 g/cm^3^), L_f_ was obtained by multiplying L_0_ by previously determined muscle length to fiber length ratio (DIA = 1; EDL = 0.44) [5,22,24,26].

#### 2.2.5. Muscle Histology

Serial cross-sections (8–10 µm thick) from each frozen left DIA and GC muscles were transversally cut into a cryostat microtome set at −20 °C (HM 525 NX, Thermo Fisher Scientific, Waltham, MA, USA) and slides (Superfrost™ Plus, Thermo Fisher Scientific) from each muscle were stained. Classical histological hematoxylin and eosin staining (H&E; Bio—Optica, Milan, Italy) was used to evaluate dystrophic muscles architecture and calculate the area of damage and regeneration (which include necrosis, inflammation, non-muscle areas, centronucleated fibers) on the total area of muscle cross-section, according to a validated protocol (TREAT—NMD SOP (ID) Number: DMD_M.1.2.007) [29]. The morphological features of the muscles were identified using digital images, acquired with a bright-field microscope (CX41, Olympus, Rozzano, Italy) and an image capture software (ImageJ, Olympus). Morphometric analysis was performed by using ImageJ analysis software on the entire muscle section, randomly selecting approximately a number of six fields per section at 10× magnification for muscle damage and 12 fields per section at 20× magnification to count centronucleated and peripherally nucleated myofibers [5,16,22].

#### 2.2.6. Spectrophotometric Determination of CK and LDH Plasma Levels

The enzymatic activity of CK and LDH in plasma samples (U/L) was determined using specific commercially available diagnostic kits (CK NAC LR and LDH LR, SGM, Rome, Italy). Both the assays required the use of a spectrophotometer (Ultrospec 2100 Pro UV/Visible, Amersham Biosciences, UK) set to a wavelength of 340 nm, at 37 °C and were performed according to manufacturer’s instructions [5,16,25,27].

### 2.3. Statistical Analysis

All experimental data were expressed as mean ± standard error of the mean (SEM). Multiple statistical comparisons between groups (WT, *mdx* + vehicle, *mdx* + DAS/HP-β-CD 10% 10 or 20 mg/kg) were performed by one-way analysis of variance (ANOVA), with Bonferroni’s *t*-test post hoc correction when the null hypothesis was rejected (*p* < 0.05). This allowed the evaluation of intra- and inter-group variability, as well as inter-group statistical comparison, while controlling the experiment-wise error rate for false positive (type I error). Data presented herein follow with good approximation a normal distribution, being included in the 95% confidence interval of the mean; this generally allows to clearly identify outliers, if any, and to apply the statistical analysis described above. No outliers were found during this study; missing data in the results were then related only to overt technical issues during the experimental procedures which led to exclude those specific samples from the analysis [27]. Whenever possible, the recovery score, an objective index that directly indicates how much of the deficit is recovered (%) by a treatment [5], was calculated for quantitatively measurable outcomes according to TREAT-NMD (SOP (ID) Number: DMD_M.1.1.001), as follows:

(1) $$\mathrm{Recovery}\;\mathrm{score}=\frac{\left(\mathrm{treated}\;\mathrm{HU}\;\mathrm{mice}-\mathrm{untreated}\;\mathrm{HU}\;\mathrm{mice}\right)}{\left(\mathrm{control}\;\mathrm{mice}-\mathrm{untreated}\;\mathrm{mice}\right)}\times 100$$

## 3. Results

### 3.1. DAS/HP-β-CD 10% Oral Complex Distribution in Plasma and Tissues of mdx Mice

**Figure 1:**
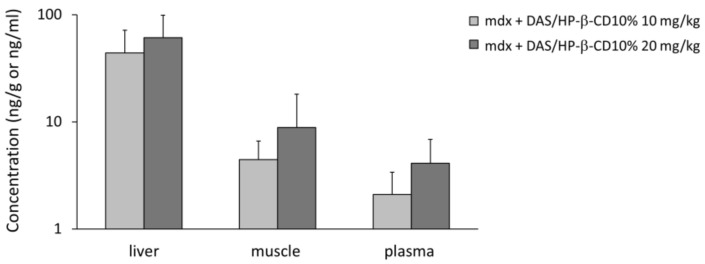
The histogram shows the concentration of dasatinib detected in tissue (liver and quadriceps muscle, ng/g) and plasma samples (ng/mL) from *mdx* mice treated with DAS/HP-β-CD 10% oral formulation at the doses of 10 and 20 mg/kg. Values are expressed as mean ± SEM from n = 9 samples at 10 mg/kg and n = 8 samples at 20 mg/kg for each type of matrix. A base-10 log scale is used for the Y axis.

After the chronic treatment with the new DAS/HP-β-CD 10% oral complex, DAS was clearly detectable in plasma samples (ng/mL) and in QUAD muscle and liver (ng/g) of *mdx* animals, at both doses (10 and 20 mg/kg), with the following order of concentration: liver > QUAD > plasma (Figure 1). This is in line with previously published observations that dasatinib rapidly distributes in tissues; also, the levels reached in skeletal muscle are in a concentration range allowing its pharmacological action to take place [16,17,30]. In all three matrices, the proportionality between the two doses was reflected, despite the high variability within each group.

### 3.2. Effect of DAS/HP-β-CD 10% on β-DG Protein Levels in mdx DIA and GC Muscles

**Figure 2:**
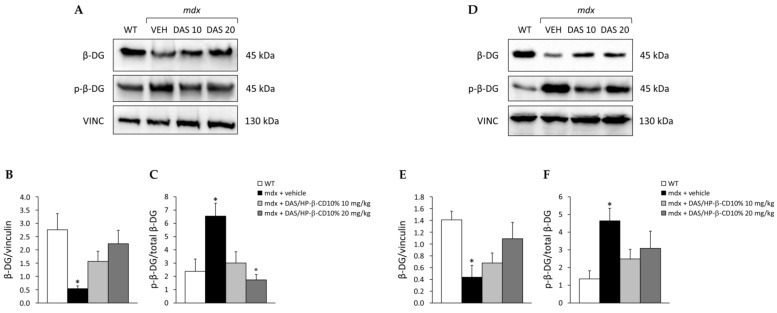
In (**A**,**D**) are shown representative Western blots for total β-dystroglycan (β-DG), phosphorylated (p-) β-DG, and reference standard vinculin (VINC) proteins, carried out in diaphragm (DIA) and gastrocnemius (GC) muscles, respectively. Total β-DG levels on VINC, and p-β-DG/β-DG ratio, are represented in (**B**,**C**) for DIA, and in (**E**,**F**) for GC muscle. Values are expressed as mean ± SEM obtained from WT mice (n = 4–6), and *mdx* mice treated with vehicle (VEH; n = 5–7), or DAS/HP-β-CD 10% at the dose of 10 (n = 7–9) or 20 (n = 6–8) mg/kg. For DIA muscle, a statistically significant difference among groups was found by one-way ANOVA for β-DG/vinculin (**B**); F = 3.6, *p* < 0.03 and p-β-DG/β-DG ratio (**C**); F = 5.5, *p* < 0.007. Bonferroni post hoc test for individual differences between groups is as follows: * vs. WT (*p* < 0.03), ° vs. *mdx* + vehicle (*p* < 0.006). For GC muscle, a statistically significant difference among groups was found by one-way ANOVA for β-DG/vinculin (**E**); F = 3.8, *p* < 0.02 and *p*-β-DG/β-DG ratio (**F**); F = 3.2, *p* < 0.05. Bonferroni post hoc test for individual differences between groups is as follows: * vs. WT (*p* < 0.05); N.S. vs. *mdx* + vehicle (*p* > 0.05).

The ability of the novel formulation to restore β-DG expression and decrease its phosphorylated form in dystrophic skeletal muscles was assessed by Western blot (Figure 2). In detail, we measured β-DG levels (expressed as ratio on reference standard, vinculin) and the p-β-DG/β-DG ratio in DIA (Figure 2A–C) and GC (Figure 2D–F) muscles. Untreated *mdx* mice showed a significant reduction of β-DG levels in both muscles (Figure 2B,E), paralleled by a significant increase in p-β-DG/β-DG ratio (Figure 2C,F) with respect to WT mice. The 12-week treatment with dasatinib induced a clear trend toward increment of β-DG levels in either DIA (Figure 2B) or GC (Figure 2E) muscle, with the 20 mg/kg dose being more efficient in restoring β-DG protein expression, as shown by the recovery scores (10 mg/kg: 46% for DIA, 25% for GC; 20 mg/kg: 76% for DIA, 67% for GC). In DIA muscle, the drug was also effective in reducing phosphorylated β-DG form at both doses (recovery score: 85% and 116%, respectively), with a significant decrease in mice treated with the 20 mg/kg dose vs. untreated ones (Figure 2C). A similar trend was observed in GC muscle, with a milder reduction in p-β-DG/β-DG ratio at both doses (recovery score: 66% and 47%, respectively) (Figure 2F).

### 3.3. Effect of DAS/HP-β-CD 10% on In Vivo Readouts in Dystrophic Mice

**Figure 3:**
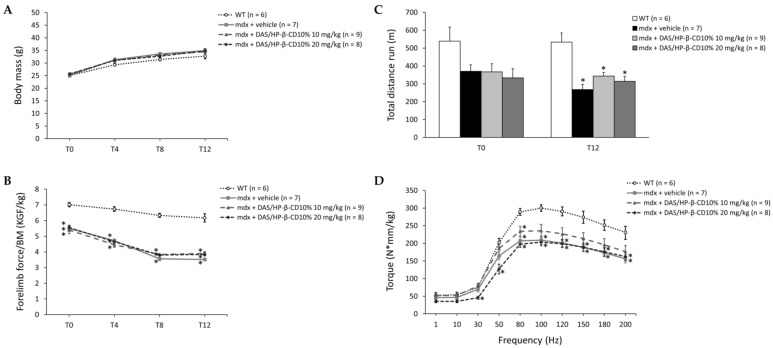
In (**A**,**B**) are shown the variations in body mass (BM, g; (**A**)) and maximal forelimb grip strength normalized to BM (KGF/kg; (**B**)) at time points T0, T4, T8, and T12 for all mice cohorts (WT mice, and *mdx* mice treated with vehicle, or DAS/HP-β-CD 10% at the dose of 10 or 20 mg/kg). Values are expressed as mean ± SEM from the number of mice indicated in brackets. For (**B**), a statistically significant difference among groups was found by one-way ANOVA at all time points (F > 12.81, *p* < 0.0001). Bonferroni post hoc test for individual differences between groups is as follows: * vs. WT (0.0001 < *p* < 0.0002); N.S. vs. *mdx* + vehicle (*p* > 0.05). In (**C**) are shown the results from an exhaustion test on the treadmill, expressed as total distance run (m) during the test carried out both at T0 and T12 on all mice cohorts. Values are expressed as mean ± SEM from the number of mice indicated in brackets. A statistically significant difference among groups was found by one-way ANOVA at T12 (F = 12, *p* < 0.0001). Bonferroni post hoc test for individual differences between groups is as follows: * vs. WT (0.0001 < *p* < 0.002); N.S. vs. *mdx* + vehicle (*p* > 0.05). In (**D**) are shown the values of hind limb plantar flexor torque (N*mm/kg) produced at increasing stimulation frequencies (from 1 to 200 Hz), obtained in anesthetized mice from each cohort at T12. Values are expressed as mean ± SEM from the number of mice indicated in brackets. A statistically significant difference among groups was found by one-way ANOVA in the range of frequencies from 30 to 200 Hz (F > 5.04, *p* < 0.007). Bonferroni post hoc test for individual differences between groups is as follows: * vs. WT (0.0001 < *p* < 0.04); N.S. vs. *mdx* + vehicle (*p* > 0.05).

The 12-week drug treatment did not affect the physiological growth of mice: as expected, all animals showed an age-dependent increase in BM at the main time points (T0, T4, T8, and T12), with a BM value higher in *mdx* vs. WT mice (Figure 3A). Forelimb grip strength was normalized to each mouse BM to avoid any bias due to intra- and inter-genotype differences. As shown in Figure 3B, all *mdx* mice cohorts were significantly weaker compared to WT, with a slight improvement in forelimb force of dystrophic mice treated with both drug doses at the end of the treatment (recovery score at T12: 14% and 12% for DAS/HP-β-CD 10% 10 and 20 mg/kg, respectively).

In vivo resistance to exercise was assessed at T0 and T12 by means of an exhaustion test on the treadmill. At T0, all *mdx* mice groups exhibited a decrease around −35% in total distance run with respect to WT mice, which became statistically significant at T12. Dasatinib partially counteracted *mdx* mice in vivo fatigability, with a recovery score of 28% and 17% at the lower and at the higher dose, respectively (Figure 3C).

Another in vivo index related to neuromuscular function is isometric plantar flexor torque, also measured at T12. As expected, untreated *mdx* mice exhibited significantly lower torque–frequency curves with respect to WT mice, particularly at higher frequencies (from 80 to 200 Hz). Dasatinib 10 mg/kg induced a trend toward amelioration of the curve, showing torque values no more statistically different from those of WT (with recovery scores ranging from to 27 to 91%), whilst the 20 mg/kg dose did not modify this parameter in *mdx* mice (Figure 3D).

**Figure 4:**
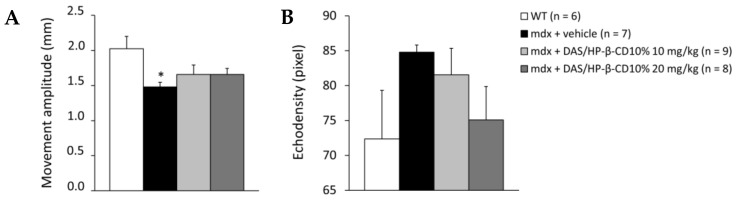
The histograms show diaphragm movement amplitude (mm; (**A**)) and pixel echodensity (**B**), measured by ultrasonography performed at T12 on all mice cohorts (WT mice, and *mdx* mice treated with vehicle, or DAS/HP-β-CD 10% at the dose of 10 or 20 mg/kg). Values are expressed as mean ± SEM from the number of mice indicated in brackets. For (**A**), a statistically significant difference among groups was found by one-way ANOVA (F = 3.1, *p* = 0.043). Bonferroni post hoc test for individual differences between groups is as follows: * vs. WT (*p* < 0.04); N.S. vs. *mdx* + vehicle (*p* > 0.05).

Results from DIA ultrasonography, assessed at T12, are shown in Figure 4. DIA movement amplitude was significantly reduced in *mdx* mice with respect to WT. DAS/HP-β-CD 10% modestly increased this index in dystrophic animals, at either dose (Figure 4A). In line with our previous results [26], no significant change in mean pixel echodensity was observed between WT and *mdx* mice at this age, with a +25% trend to increase in dystrophic animals. Again, the drug did not significantly modify this parameter, although a more pronounced reduction in echodensity was found in mice treated with the 20 mg/kg dose (Figure 4B).

### 3.4. Effects of DAS/HP-β-CD 10% on Ex Vivo Contraction of mdx DIA and EDL Muscles

**Figure 5:**
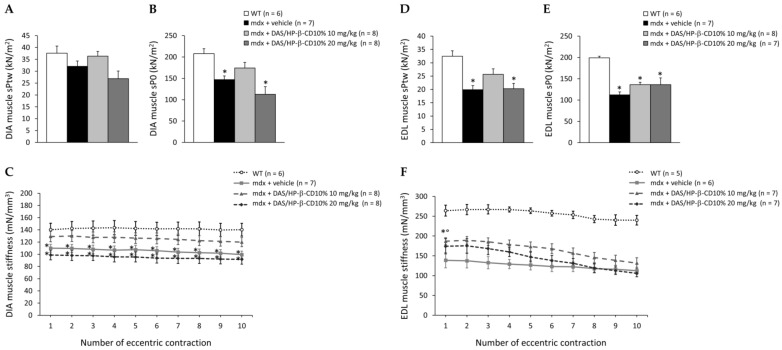
In (**A**–**C**) are shown ex vivo maximal specific isometric twitch (sPtw, kN/m^2^; (**A**)) and tetanic (sP0, kN/m^2^; (**B**) force values, and elastic properties in response to a series of 10 eccentric pulses (stiffness, mN/mm^3^; (**C**)), obtained in DIA muscle for all mice cohorts (WT mice, and *mdx* mice treated with vehicle, or DAS/HP-β-CD 10% at the dose of 10 or 20 mg/kg). Values are expressed as mean ± SEM from the number of mice indicated in brackets. A statistically significant difference among groups was found by one-way ANOVA for both (**A**) (F = 3.4, *p* = 0.03) and (**B**) (F = 8.3, *p* = 0.0005). Bonferroni post hoc test for individual differences between groups is as follows: * vs. WT (0.0004 < *p* < 0.04); N.S. vs. *mdx* + vehicle (*p* > 0.05). For (**C**), a statistically significant difference in muscle stiffness among groups was found at each pulse by one-way ANOVA (F > 4.87, *p* < 0.008). Bonferroni post hoc test for individual differences between groups is as follows: * vs. WT (0.002 < *p* < 0.05); N.S. vs. *mdx* + vehicle (*p* > 0.05). For EDL muscle, the same parameters are shown in (**D**) (sPtw), (**E**) (sP0), and (**F**) (stiffness). Values are expressed as mean ± SEM from the number of mice indicated in brackets for each experimental group. A statistically significant difference among groups was found by one-way ANOVA for both (**D**) (F = 8.5, *p* = 0.0005) and (**E**) (F = 14.6, *p* < 0.0001). Bonferroni post hoc test for individual differences between groups is as follows: * vs. WT (0.0001 < *p* < 0.001); N.S. vs. *mdx* + vehicle (*p* > 0.05). For (**F**), statistically significant difference in muscle stiffness among groups was found at each pulse by one-way ANOVA (F > 13.52, *p* < 0.0001). Bonferroni post hoc test for individual differences between groups is as follows: * vs. WT (0.0001 < *p* < 0.0007); ° vs. *mdx* + vehicle (0.0009 < *p* < 0.01).

At sacrifice, isometric and eccentric contraction recordings of isolated DIA and EDL muscles, primary readouts for assessing drug efficiency, were carried out (Figure 5). DIA muscle isometric twitch (sPtw) and tetanic (sP0) force values were lower in untreated *mdx* mice compared to those of WT mice, and this reduction was statistically significant for sP0. A partial recovery of both sPtw and sP0 was found in mice treated with 10 mg/kg DAS/HP-β-CD 10% (recovery score: 77% for sPtw and 45% for sP0) (Figure 5A,B). The 10 mg/kg dose was also partially effective in ameliorating DIA muscle compliance to stretch in response to eccentric contractions, which was severely compromised in untreated *mdx* mice, with recovery scores ranging from 50% to 63%; no benefit was exerted by the 20 mg/kg dose on these indices (Figure 5C).

As shown in Figure 5D,E, EDL muscle sPtw and sP0 were significantly reduced in *mdx* mice, as well. A partial amelioration was induced by drug treatment at both doses particularly for sP0 (recovery score: 28%, at both doses). In addition, EDL muscles from all *mdx* mice groups were significantly less compliant to stretch compared to WT. However, also in this case, a partial recovery was observed in *mdx* mice treated with the 10 mg/kg dose, showing significantly higher values with respect to untreated ones at eccentric pulses from 1 to 6, with recovery scores ranging from 15 to 40% (Figure 5E). No significant drug effects were observed in terms of single twitch kinetics, and force drop or recovery after the eccentric contraction protocol for either DIA or EDL muscle (Supplementary Table S1).

### 3.5. Effects of DAS/HP-β-CD 10% on Histopathology of mdx DIA and GC Muscles

**Figure 6:**
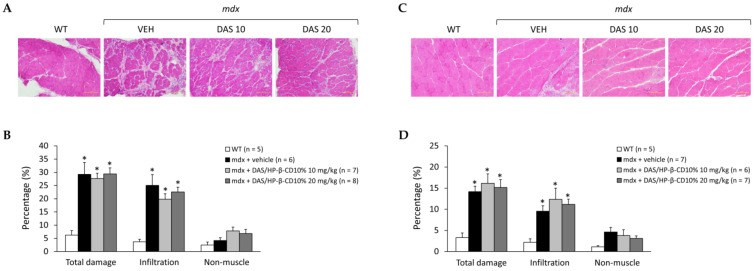
In (**A**,**C**) are shown representative DIA (**A**) and GC (**C**) muscle sections stained with hematoxylin and eosin (20× magnification) from mice of each experimental group (WT mice, and *mdx* mice treated with vehicle—VEH—or DAS/HP-β-CD 10% at the dose of 10 or 20 mg/kg). This staining allows to appreciate the organization of skeletal muscle architecture and its typical alterations in dystrophin-deficient muscles, including the presence of abnormal inflammatory infiltrates or non-muscle (i.e., fibrotic and adipose tissue) areas, quantified via subsequent morphometric analysis, as shown by histograms in (**B**) for DIA and (**D**) for GC muscle. Values are expressed as mean ± SEM from the number of mice indicated in brackets. (**B**) A statistically significant difference among groups was found by one-way ANOVA for total damage (F = 11, *p* = 0.0002) and infiltration (F = 10, *p* = 0.0003). Bonferroni post hoc test for individual differences between groups is as follows: * vs. WT (0.0002 < *p* < 0.004); N.S. vs. *mdx* + vehicle (*p* > 0.05). (**D**) A statistically significant difference among groups was found by one-way ANOVA for total damage (F = 6.5, *p* = 0.003) and infiltration (F = 4.2, *p* = 0.02). Bonferroni post hoc test for individual differences between groups is as follows: * vs. WT (0.006 < *p* < 0.04); N.S. vs. *mdx* + vehicle (*p* > 0.05).

The potential effect of the novel formulation on histopathology was assessed on H&E-stained DIA and GC muscle sections, whose representative images are shown in Figure 6A,C, respectively. As expected, the typical hallmarks of dystrophic histopathology were clearly present in both muscle types, characterized by muscle architecture alteration, with the presence of necrotic areas, inflammatory infiltrates, and non-muscle areas. The presence of centronucleated myofibers, index of cyclic degeneration–regeneration events, was also clearly detectable. However, a great variability within different mice and within different fields of the same muscle was observed, which required a detailed morphometric analysis to clearly appreciate any possible difference among mice groups.

As shown in Figure 6B, DIA muscle of untreated *mdx* mice showed a significantly higher percentage of total area of damage, and specifically of inflammatory cell infiltrates, in comparison to WT. The drug did not ameliorate DIA histopathology at any dose. Results in GC muscle were almost overlapping (Figure 6D). Furthermore, the drug did not reduce the high percentage of centronucleated fibers found in either DIA or GC muscles from *mdx* animals (Table 1).

### 3.6. Effects of DAS/HP-β-CD 10% on Hind Limb Muscles and Vital Organs Mass, and Plasma Biomarkers of Muscle Damage

The masses of main hind limb muscles (TA, EDL, QUAD, GC, SOL) and vital organs (liver, heart, kidneys, spleen, brain), normalized to each mouse BM (mg/g), are shown in Table 2. A significant increase in liver mass was found in all *mdx* groups compared to WT, irrespective of drug treatment, and coherently with our previous observations in this mouse model [5,22,25]. No variations between the experimental groups were found for other vital organs. A significant increment in TA, GC, and QUAD muscles mass was found in *mdx* mice vs. WT, with no effects of drug treatment (Table 1).

**Figure 7:**
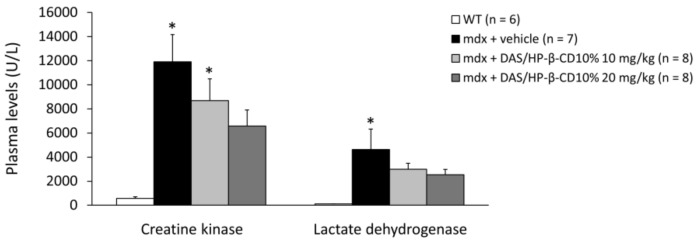
The histogram shows the levels of enzymes creatine kinase (CK) and lactate dehydrogenase (LDH) (U/L), measured in plasma samples collected from mice of each experimental group (WT mice, and *mdx* mice treated with vehicle, or DAS/HP-β-CD 10% at the dose of 10 or 20 mg/kg). Values are expressed as mean ± SEM from the number of mice indicated in brackets. A statistically significant difference among groups was found by one-way ANOVA for CK (F = 7.4, *p* = 0.001) and LDH (F = 3.8, *p* = 0.02). Bonferroni post hoc test for individual differences between groups is as follows: * vs. WT (0.0007 < *p* < 0.02); N.S. vs. *mdx* + vehicle (*p* > 0.05).

Plasma levels (U/L) of CK and LDH enzymes are shown in Figure 7. Both enzymes were significantly increased in untreated dystrophic mice with respect to WT. The 12-week treatment with DAS/HP-β-CD 10% complex was able to reduce, although non-significantly, the levels of both CK and LDH in *mdx* mice, and this was more evident with the 20 mg/kg dose, as shown by the recovery scores (10 mg/kg: 28% for CK, 36% for LDH; 20 mg/kg: 47% for CK, 46% for LDH).

## 4. Discussion

Repurposing existing drugs with well-known safety profile and pathways of action, holds great promise for a quick development of efficacious treatment for patients suffering from progressive, life-threatening diseases, as Duchenne muscular dystrophy (DMD). The potent anticancer agent dasatinib (DAS) has recently gained interest for its possible repurposing in children with DMD, in virtue of its ability to prevent tyrosine phosphorylation and degradation of β-DG, via inhibition of ROS-activated cSrc TK.

Already approved by FDA to treat pediatric CML, DAS is currently available in pharmaceutical form of film-coated tablets, whose administration may be difficult in the view of a life-long treatment of patients progressively manifesting oropharyngeal muscle weakness and dysphagia [31]. Our long-term (12-week) study in *mdx* mice was aimed to define the potential efficacy and safety of a new, pediatric-suitable, water-soluble DAS/HP-β-CD oral complex, after promising results obtained in healthy mice [17]; in parallel, we sought to confirm DAS mechanism of action seen in dystrophic muscles following s.c. administration [16].

In agreement with previous and preliminary assessment in WT mice [16,17,30], our analysis corroborated the ability of the novel oral DAS complex to efficiently distribute in *mdx* mice plasma and tissues. Plus, drug exposure in skeletal muscle at both tested doses (10 and 20 mg/kg) was in an adequate range to potentially allow its therapeutic action, that is known to occur in the nanomolar range [32].

Furthermore, in line with our proof-of-concept study [16], we confirmed that DAS, also in its new pharmaceutical form, was clearly effective in restoring total β-DG protein levels in both *mdx* DIA and GC muscles. In parallel, DAS decreased β-DG phosphorylation, thus targeting the key event responsible for β-DG degradation. Importantly, both pharmacokinetics and pharmacodynamics data evidenced that DAS/HP-β-CD oral formulation exerted its actions in a dose-proportional manner.

Therefore, we adopted a clinically oriented, multidisciplinary approach to assess DAS/HP-β-CD complex effects on validated, primary disease-relevant readouts in *mdx* mice [19,20]. Surprisingly, the new drug formulation showed a modest positive impact on in vivo indices of *mdx* neuromuscular function (i.e., forelimb force, plantar flexor torque, fatigability), with no statistically significant differences in comparison to vehicle-treated *mdx*. Likewise, a limited efficacy was observed on ex vivo DIA and EDL muscle-specific parameters related to force, as well as on histopathology; this happened irrespectively of the dose administered and in spite of the clear effects of DAS on β-DG protein and elasticity.

Although modest, it was interesting to observe a DAS effect on muscle stiffness, which paralleled its ability to partially reduce high plasma levels of CK and LDH in *mdx* mice, similarly to what was formerly observed in our proof-of-concept study [16]. Both these effects could be indices of an improved sarcolemmal stability, likely related to the main mechanism of action and to the increase in β-DG expression, suggesting DAS potential to structurally reinforce the muscle. The observed modest global effectiveness of DAS matches with our previous observations following the short-term subcutaneous treatment in *mdx* mice and pushes towards wider considerations.

A likely hypothesis for the scarce overall efficacy of DAS in *mdx* mice, also in a better formulation and over a long-term, could be that, despite the increase of β-DG levels in skeletal muscle fibers, the protein may be not sufficiently expressed at the sarcolemma. In fact, considering the direct interactions of dystrophin and β-DG at the membrane level in normal muscle fibers [13,33], the stability of β-DG at sarcolemma remains weak or incorrectly localized in dystrophin-lacking fibers, in turn limiting DAS effects at the functional and structural level. In this view, further insights will be gained by means of dedicated immunofluorescence and molecular biology experiments. In addition, it would be interesting to distinguish the localization at either sarcolemmal or nuclear membrane, since it has been reported that β-DG, albeit in small amounts, is localized at the nucleus in myoblasts [34].

Another possible reason accounting for the limited efficacy observed on main dystrophy-related outcomes, could be that DAS, apart from its main action on β-DG, was ineffective in controlling multiple cSrc-TK-mediated downstream damaging events (e.g., inflammation, oxidative stress, autophagy). Since DAS is a pan-inhibitor of several TK families [30,35], this might have masked the potential benefit directly correlated to the inhibition of cSrc-TK, considering the housekeeping role of this family of kinases in various tissues, including skeletal muscle [36]. In this regard, it is worth mentioning that more promising results were obtained with the more selective inhibitor PP2 [16], which was not included in this study, being not an approved drug.

Importantly, the gross examination of vital organs’ mass allowed us to exclude any possible drug-induced toxicity which could have hampered the possible benefits of the treatment in dystrophic animals. Moreover, our published in vitro results from cytotoxicity assays in C2C12 cells, showed that DAS was toxic only at high concentrations, largely exceeding those reached in response to in vivo exposure [16], and possibly occurring for a metabolic toxicity as recently showed by Bouitbir et al. [37]. Our interest in metabolic and mitochondrial dysfunction in dystrophic muscles pushes toward a more detailed assessment for a possible greater susceptibility to DAS actions which contribute to hinder functional recovery.

## 5. Conclusions

We tested a brand new oral formulation of dasatinib complexed with cyclodextrin in *mdx* mice, which allowed a good chronic drug exposure, without toxicity, at different doses (10 and 20 mg/kg). DAS confirmed its main upstream mechanism of action, by increasing non-phosphorylated β-DG protein levels in dystrophic muscles, although it was not sufficient to significantly improve the main in vivo and ex vivo clinically relevant readouts in *mdx* mice. In this context, the association of oral DAS formulation with therapeutics based on stop codon read-through approach, such as aminoglycoside antibiotics (e.g., gentamicin) and orally available small molecules (e.g., ataluren), could be useful for their potential synergistic action on the restoration of the DGC complex and, in turn, on the DMD pathogenic cascade [38]. Additionally, the association of DAS with standard glucocorticoids, as an alternative to, or in combination with, synergistic aminoglycosides and compounds targeting nonsense mutations, could possibly help to better control chronic inflammation and fibrosis in DMD patients.

As aforementioned, DAS is a highly promiscuous kinase inhibitor [30]. It is reasonable to consider that its actions on other TKs may have hidden its therapeutic effects in our model. This will be further investigated via ad hoc analyses in a follow-up study; importantly, this latter will also include the measurement of the levels of Src tyrosine kinase and its phosphorylated form, as potentially relevant data to correlate with the doses applied in the study. In addition, dedicated in silico and in vitro studies will be performed for the identification of novel selective cSrc TK inhibitors to be potentially tested in preclinical studies for DMD.

## Figures and Tables

**Table 1:** The table shows the percentage of centronucleated fibers, quantified via qualitative analysis on H&E stained sections of diaphragm (DIA) and gastrocnemius (GC) muscles (10× magnification) from all mice cohorts. Values are expressed as mean ± SEM from the number of mice indicated in brackets. A statistically significant difference among groups was found by one-way ANOVA for both DIA (F = 7.5, *p* = 0.002) and GC (F = 64, *p* = 0.0001). Bonferroni post hoc test for individual differences between groups is as follows: * vs. WT (0.0001 < *p* < 0.002); N.S. vs. *mdx* + vehicle (*p* > 0.05).

Group	Centronucleated Fibers (%)	
	DIA	GC
WT (n = 5)	1.02 ± 0.32	1.95 ± 0.44
*mdx* + vehicle (n = 5)	26.1 ± 2.7 *	68.2 ± 3.4 *
*mdx* + DAS/HP-β-CD10% 10 mg/kg (n = 7)	23.6 ± 4.7 *	76.1 ± 2.7 *
*mdx* + DAS/HP-β-CD10% 20 mg/kg (n = 8)	21.1 ± 2.9 *	74.3 ± 3.7 *

**Table 2:** The table shows the mass of hind limb tibialis anterior (TA), extensor digitorum longus (EDL), quadriceps (QUAD), gastrocnemius (GC), and soleus (SOL) muscles, and the mass of vital organs (liver, heart, kidneys, spleen, and brain), normalized to mice body mass (BM), in mg/g, for all the experimental groups. Values are expressed as mean ± SEM from the number of mice indicated in brackets. A statistically significant difference among groups was found by one-way ANOVA for TA (F = 20.06, *p* < 0.0001), QUAD (F = 16.22, *p* < 0.0001), GC (F = 10.73, *p* < 0.0001), and SOL (F = 3.08, *p* = 0.05), and for vital organs, as well as for liver (F = 5.98, *p* = 0.003) and brain (F = 6.2, *p* = 0.003). Bonferroni post hoc test for individual differences between groups is as follows: * vs. WT (0.0001 < *p* < 0.05); N.S. vs. *mdx* + vehicle (*p* > 0.05).

Group	Mass of Hind Limb Muscles/BM (mg/g)					Mass of Vital Organs/BM (mg/g)				
	TA	EDL	QUAD	GC	SOL	Liver	Heart	Kidneys	Spleen	Brain
WT (n = 6)	1.57 ± 0.07	0.60 ± 0.22	6.72 ± 0.57	5.34 ± 0.15	0.27 ± 0.02	42.2 ± 1.5	4.78 ± 0.17	6.71 ± 0.15	2.81 ± 0.10	13.6 ± 0.23
*mdx* + vehicle (n = 7)	2.30 ± 0.10 *	0.44 ± 0.01	9.95 ± 0.37 *	6.36 ± 0.11 *	0.34 ± 0.03	49.8 ± 1.4 *	4.79 ± 0.11	6.41 ± 0.08	3.03 ± 0.09	11.9 ± 0.67
*mdx* + DAS/HP-β-CD10% 10 mg/kg (n = 8)	2.27 ± 0.06 *	0.37 ± 0.02	9.86 ± 0.20 *	6.14 ± 0.11 *	0.37 ± 0.03	47.7 ± 1.0 *	4.78 ± 0.12	6.89 ± 0.53	3.13 ± 0.08	12.1 ± 0.32
*mdx* + DAS/HP-β-CD10% 20 mg/kg (n = 8)	2.23 ± 0.06 *	0.37 ± 0.02	9.48 ± 0.32 *	6.21 ± 0.16 *	0.39 ± 0.03	47.8 ± 1.3 *	5.01 ± 0.11	6.37 ± 0.11	2.97 ± 0.10	11.4 ± 0.39

## Data Availability

The data presented in this study are available on request from the corresponding author. Some data may not be made available because of privacy or ethical restrictions.

## Supplementary Materials

The following are available online at https://www.mdpi.com/article/10.3390/biom11111742/s1, Table S1: Additional contractile parameters of isolated DIA and EDL muscles.
