# Supplementary material for: β-Dystroglycan Restoration and Pathology Progression in the Dystrophic mdx Mouse: Outcome and Implication of a Clinically Oriented Study with a Novel Oral Dasatinib Formulation
Source: Biomolecules. 2021 Nov 22;11(11):1742. doi: 10.3390/biom11111742 (PMC8615430; doi:10.3390/biom11111742)
Supplement: Supplementary file 1 [file biomolecules-11-01742-s001.zip › biomolecules-1429162-supplementary.pdf]

Group	DIA				EDL			
	TTP (ms)	HRT (ms)	Force drop after eccentric protocol (%)	Recovery 30 min after eccentric protocol (%)	TTP (ms)	HRT (ms)	Force drop after eccentric protocol (%)	Recovery 30 min after eccentric protocol (%)
WT (n = 6)	30.2 ± 1.8	18.1 ± 0.54	- 1.8 ± 1.3	- 3.7 ± 2.0	19.7 ± 0.7	18.9 ± 0.41	- 35.6 ± 3.8	- 7.1 ± 2.7
mdx + vehicle (n = 7)	33.2 ± 1.3	21.3 ± 0.80	- 12.9 ± 2.0*	- 13.1 ± 1.4*	22.1 ± 2.1	16.3 ± 0.93	- 55.3 ± 5.5	- 28.9 ± 3.1
mdx + DAS/HP-β-CD10% 10 mg/kg (n = 8)	31.8 ± 1.5	18.9 ± 0.53	- 14.1 ± 0.70*	- 16.3 ± 1.3*	22.7 ± 1.2	18.1 ± 0.56	- 56.6 ± 4.7	- 19.1 ± 4.3
mdx + DAS/HP-β-CD10% 20 mg/kg (n = 8)	30.5 ± 1.1	20.8 ± 1.33	- 17.5 ± 4.2*	- 16.5 ± 2.6*	21.6 ± 1.0	15.4 ± 0.32	- 59.4 ± 6.9	- 35.9 ± 7.6*

Table S1. The table shows single isometric twitch contraction kinetic parameters (time to peak, TTP; half relaxation time, HRT), the percentage of isometric tetanic force drop induced by eccentric contractions (a series of 10 stimuli, every 30 s), and the percentage of tetanic force recovery 30 min after the eccentric protocol, obtained in diaphragm (DIA) and extensor digitorum longus (EDL) muscles from all mice cohorts. Values are expressed as mean ± SEM from the number of mice indicated in brackets. For DIA, a statistically significant difference among groups was found by one-way ANOVA for force drop at the 10th eccentric pulse ($F = 6.3$, $p = 0.003$) and force recovery after 30 min ($F = 9.3$, $p = 0.0003$). For EDL, a statistically significant difference among groups was found by one-way ANOVA for force drop at the 10th eccentric pulse ($F = 3.3$, $p < 0.04$) and force recovery after 30 min ($F = 5.2$, $p = 0.007$). Bonferroni post hoc test for individual differences between groups is as follows: * vs. WT ($0.0001 < p < 0.008$); N.S. vs. *mdx* + vehicle ($p > 0.05$).
